# MIF inhibitor, ISO-1, attenuates human pancreatic cancer cell proliferation, migration and invasion *in vitro*, and suppresses xenograft tumour growth *in vivo*

**DOI:** 10.1038/s41598-020-63778-y

**Published:** 2020-04-21

**Authors:** Bo Cheng, Qiaofang Wang, Yaodong Song, Yanna Liu, Yanyan Liu, Shujun Yang, Dejian Li, Yan Zhang, Changju Zhu

**Affiliations:** Department of Emergency, the First Affiliated Hospital of Zhengzhou University, Henan, 450052 China

**Keywords:** Cancer, Drug discovery

## Abstract

This study sought to investigate the biological effects of specific MIF inhibitor, ISO-1, on the proliferation, migration and invasion of PANC-1 human pancreatic cells *in vitro*, and on tumour growth in a xenograft tumour model *in vivo*. The effect of ISO-1 on PANC-1 cell proliferation was examined using CCK-8 cell proliferation assay. The effect of ISO-1 on collective cell migration and recolonization of PANC-1 cells was evaluated using the cell-wound closure migration assay. The effect of ISO-1 on the migration and invasion of individual PANC-1 cells in a 3-dimensional environment in response to a chemo-attractant was investigated through the use of Transwell migration/invasion assays. Quantitative real time PCR and western blot analyses were employed to investigate the effects of ISO-1 on MIF, NF-κB p65 and TNF-α mRNA and protein expression respectively. Finally, a xenograft tumor model in BALB/c nude mice were used to assess the *in vivo* effects of ISO-1 on PANC-1-induced tumor growth. We found high expression of MIF in pancreatic cancer tissues. We demonstrated that ISO-1 exerts anti-cancer effects on PANC-1 cell proliferation, migration and invasion *in vitro*, and inhibited PANC-1 cell-induced tumour growth in xenograft mice *in vivo*. Our data suggests that ISO-1 and its derivative may have potential therapeutic applications in pancreatic cancer.

## Introduction

Pancreatic cancer exhibits very poor prognosis and extremely high mortality rate with a five-year survival rate of less than 5%^1–3^. As a result of its unique anatomical location, low degree of differentiation, and rapid disease development, most pancreatic malignancy presents at advances stages at the time of diagnosis^4^. Furthermore, as prominent resistance to systemic chemotherapy and radiotherapy often develops, surgical resection remains the only treatment option with curative potential^5–7^. However, less than 20% of patients are presented with this opportunity^8,9^. Despite advances in our understanding of the biology of pancreatic cancer development and progression, we are far from providing a curative answer. Therefore, there is still an urgent need for the identification and/or development of novel effective agents for the treatment of this aggressive malignancy.

Macrophage migration inhibitory factor (MIF), a member of the tautomerase family of cytokines, was originally identified as a T-cell-derived factor that inhibits the random migration of macrophages^10,11^. MIF is now recognized as a pleiotropic pro-inflammatory mediator playing critical roles in the innate and adaptive immune response and the overall inflammatory cascade^12–14^. MIF expression is elevated in numerous inflammatory and autoimmune diseases correlating positively with disease severity^15,16^. Given that inflammation and immunity are intimately associated with cancer progression, severity and unfavourable prognostic outcomes, it is not surprising that MIF expression have been found to be elevated in numerous cancers including squamous carcinoma, glioblastoma, cervical adenocarcinoma, malignant melanoma, nasopharyngeal cancer, colon cancer, lung cancer, breast cancer, and prostate cancer^17–20^. Studies have also shown high expression of MIF in pancreatic cancer^21,22^ Importantly, the expression of MIF is directly and positively correlated with the aggressiveness of the cancer phenotype. MIF have been suggested to be the “key” that links inflammation with cancer development and progression. Thus, MIF is an attractive target for the development of therapeutic inhibitors.

ISO-1 is a highly specific MIF tautomerase activity inhibitor and frequently used as the reference inhibitor for MIF^23,24^. ISO-1 treatment in mice has been shown to markedly inhibit the tumorigenic growth of prostate cancer, colon cancer, gallbladder cancer, osteosarcoma, lung adenocarcinoma, glioblastoma, and melanoma^25–27^. However, the effects of ISO-1 in pancreatic cancer remains to be examined and hence the purpose of this study. Here, using cell-based assays we showed that ISO-1 markedly inhibited the proliferation, migration and invasion of PANC-1 cells *in vitro*. By gene and protein expression analyses we further demonstrated the downregulation MIF, NF-κB p65 and TNF-α. Additionally the pNF-κB p65 was significanlty reduced suggesting inhibition of NF-κB signaling. Finally using a *in vivo* xenograft tumour model in nude mice, we showed that ISO-1 treatment resulted in significant reduction in PANC-1 cell-induced tumour growth with concomitant reduction in MIF expression in tumour tissues. Collectively, our findings demonstrates that ISO-1 may offer beneficial effects against pancreatic cancer.

## Results

### The expression of MIF is elevated in human pancreatic cancer tissues

**Figure 1:**
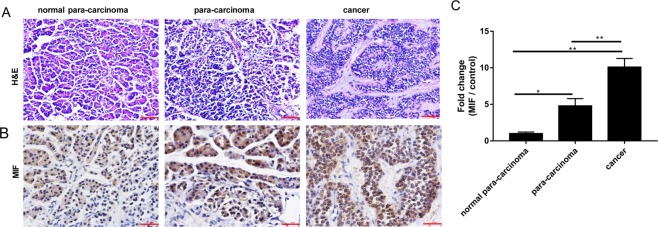
Expression of MIF in human para-carcinoma and pancreatic cancer tissues. Normal tissue, para-carcinoma tissue, and pancreatic cancer tissue were subjected to **(A)** H&E staining (magnification100×) or **(B)** MIF IHC staining (magnification 200×). **(C)** MIF expression in pancreatic cancer tissues were quantified and expressed as fold change relative to normal controls; **p < 0.01 when compared to normal pancreatic tissue.

We first reaffirmed the involvement of MIF in pancreatic cancer tumourigenesis by examining the expression of MIF in human pancreatic cancer tissue samples, in para-carcinoma tissues and in normal tissue. Tissue samples were obtained from the Department of Pathology of the First Affiliated Hospital of Zhengzhou University. As shown in Fig. 1A,B, significant elevation in MIF positive staining was observed in both para-carcinoma and pancreatic cancer tissue samples as compared to normal tissue. Semi-quantitative measurement based on the integrated optical density (IOD) of staining intensity, showed that the expression of MIF was elevated 10-folds in pancreatic cancer tissues as compared to normal tissues (Fig. 1C). This data confirms a role for MIF in pancreatic cancer tumourigenesis.

### Effects of ISO-1 on PANC-1 pancreatic cancer cell proliferation

**Figure 2:**
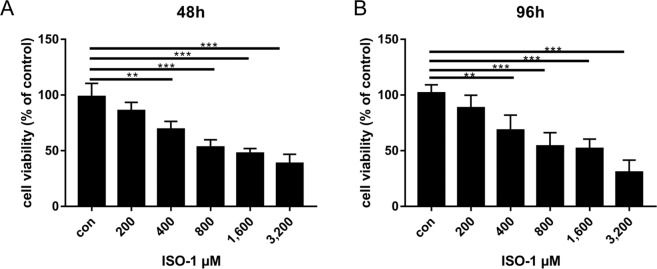
ISO-1 repressed the proliferation of PANC-1 human pancreatic cancer cells. PANC-1 cells were treated without or with serial dilutions of ISO-1 (200, 400, 800, 1600 and 3200 μM) for **(A)** 48 and **(B)** 96 hours. Graphs are presented as the mean ± SD of 5 independent experiments; **p < 0.01, ***p < 0.001 when compared with untreated controls.

We next investigated the biological significantce of the inhibition of MIF activity in PANC-1 pancreatic cancer cells using the MIF inhibitor ISO-1. The effect of ISO-1 on PANC-1 cell proliferation was first evaluated using the CCK-8 proliferation/cytotoxicity assay. Based on the reduction of the water soluble tetrazolium salt WST to formazan dye by cellular dehydrogenase, the amount of which is directly proportional to the number of live cells, we see that ISO-1 treatment dose-dependently inhibited the proliferation of PANC-1 cells at both 48 and 96 hour time points (Fig. 2A,B respectively). At both time points, 25 and 50% reduction in cell proliferation was achieved at concentrations of 400 and 800 μM respectively. Concentrations beyond 800 μM did not exert further appreciable increase in inhibitory effect. No significant effect on PANC-1 cell proliferation was observed at 200 μM. Therefore, ISO-1 (>400 μM) significantly inhibited PANC-1 cell proliferation *in vitro*.

### ISO-1 inhibited the collective migration and recolonization of PANC-1 cells

**Figure 3:**
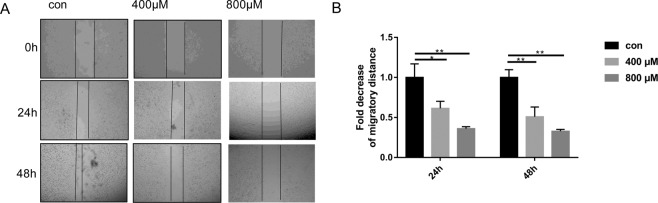
Inhibition of the collective migration and recolonization ability of PANC-1 cells by ISO-1. **(A)** Representative phase contrast images wound area when it was first generated (0), at 24 hours and at 48 hours after ISO-1 treatment; (magnification 100×). **(B)** Quantitative analysis of the cell migration at 24 and 48 hours expressed as the fold decrease in migratory distance relative to untreated controls. Graphs are presented as mean ± SD from 3 independent experiments; *p < 0.05, **p < 0.01, and ***p < 0.001 when compared with untreated control.

Cancer metastasis and progression is the main cause of death in patients, hence the investigation into the effects of therapeutics on the prevention of cancer cell migration and invasion is of utmost importance. To this end we employed the cell wound closure assay to examine the effects of ISO-1 treatment on the collective migration of PANC-1 cell mass in a 2-dimensional environment. A scratch wound was generated down the center of the confluent monolayer of PANC-1 cells after which cells were treated with indicated concentrations of ISO-1 for 24 and 48 hours (Fig. 3A). The migratory distance at each time point under each treatment condition was compared to time point 0 (when the scratch wound was created) and then expressed as a fold change relative to untreated controls (Fig. 3B). As shown in Fig. 3A, untreated PANC-1 cells exhibited a time-dependent reduction in the scratch wound space between the two opposing cell fronts indicating migration and recolonization of wound area by cells. Treatment with ISO-1 on the other hand, inhibited the collective migration of the cell mass and prevented the closure of the scratch wound area. This was particularly obvious with cells treated with 400 and 800 μM ISO-1. At 48 hours after treatment, the scratch wound width was observed to be more or less the same as the scratch wound initially generated. This observation was confirmed by quantitative measurement of the migratory distance after treatment with ISO-1. The migratory distance for cells treated with ISO-1 were markedly reduced relative to untreated controls (Fig. 3B). Together these results suggest that ISO-1 inhibited the collective planar migration of PANC-1 cells in 2-dimensional environment.

### ISO-1 attenuated PANC-1 single cell migration and invasion

**Figure 4:**
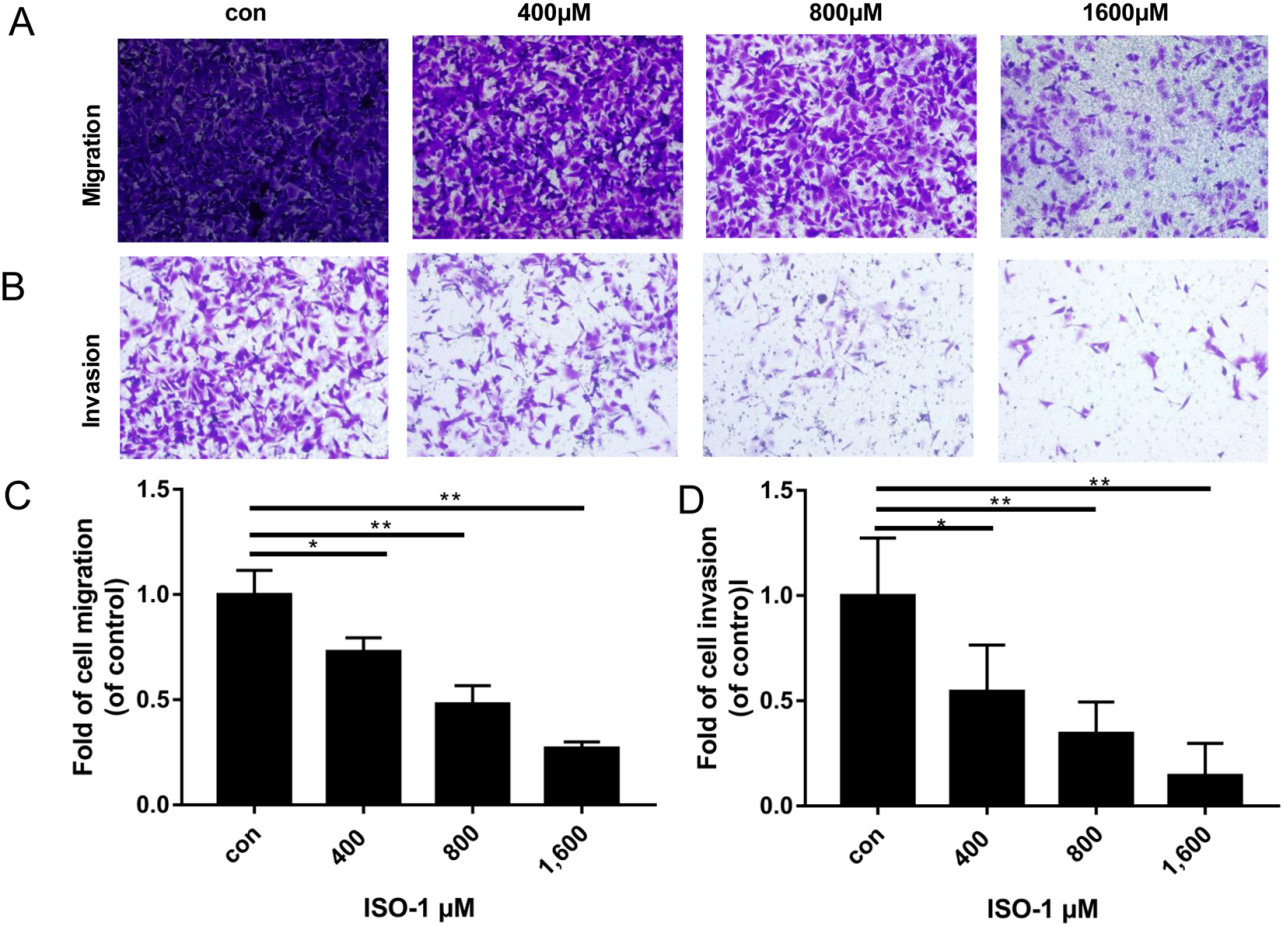
Attenuation of directional migration and invasion of PANC-1 cells following ISO-1 treatment. Representative phase contrast of crystal violet stained PANC-1 cells that **(A)** migrated through the transwell membrane or **(B)** invaded through the Matrigel Matrix after 24 hours of ISO-1 treatment (magnification 200×). **(C,D)** Quantitative analysis of PANC-1 cell migration and invasion respectively, expressed as fold change relative to untreated controls. Graphs are presented as the mean ± SD from 3 independent experiments; *p < 0.05, **p < 0.01 when compared with untreated controls.

In addition to the collective migration of the cancer cell mass, individual cancer cells must also need to demonstrate the capacity to migrate and invade through the extracellular matrix and intravasate into the circulation for colonization at distant secondary sites. Thus, we used the transwell migration chambers to assess the ability of individual PANC-1 cells to directionally migrate and invade through a 3-dimensional environment in response to a chemo-attractant. For our purpose, an FBS gradient was established (15% in bottom chambers versus 2.5% in upper transwell inserts) and used as the chemo-attractant for cell migration/invasion. Transwell chambers without or coated with Matrigel matrix was respectively used to explore the migration and invasion of PANC-1 cells in response to ISO-1 treatment. As shown in Fig. 4A,B, a dose-dependent decrease in PANC-1 cell migration and invasion through the transwell chambers was observed after 24 hours treatment with ISO-1. In terms of cell migration, significant difference was observed at 400 μM with more than 50% reduction in cell migration observed at concentration of 800 μM (Fig. 4C). At 1600 μM (1.6 mM) of ISO-1 treatment, cell migration was reduced by 75%. For PANC-1 cell invasion capacity, significant reduction in invasion was again observed at concentrations of 400 μM and above. However, 50% reduction in invasion was observed at 400 μM and at 1.6 mM of ISO-1 only around 10% of cells exhibited invasive capabilities (Fig. 4D). Collectively these results suggest that ISO-1 can attenuate the directional migration and invasion of individual PANC-1 cells towards area of higher FBS concentration (chemo-attractant).

### ISO-1 downregulated the expression of MIF, NF-κB p65 and TNF-α

**Figure 5:**
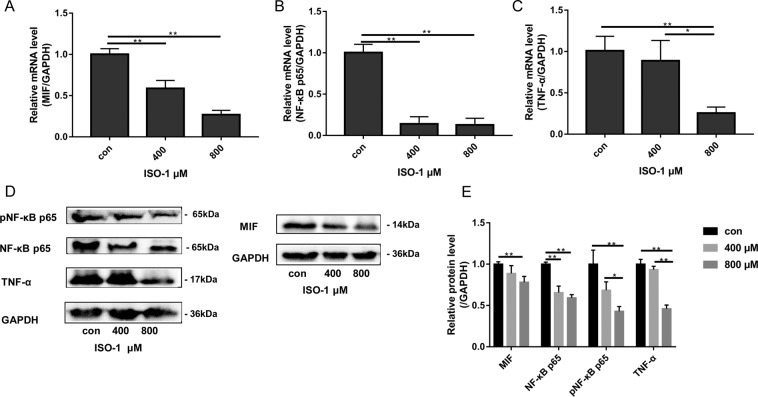
Effects of ISO-1 on the mRNA and protein expression of MIF, NF-κB p65 and TNF-α. Western blot was obtained by using gel under uniform standard conditions and by cutting the film under uniform standard exposure conditions. The film was cut according to marker and the target strip was retained. Therefore, Fig. 5 shows the cut film. High contrast (overexposed) films are not used. mRNA expression of **(A)** MIF, **(B)** NF-κB p65 and **(C)** TNF-α were examined by real-time qPCR using RNA extracted from PANC-1 cells treated without or with indicated concentrations of ISO-1 for 24 hours. **(D)** Western blot analyses were conducted on protein lysates extracted from PANC-1 cells treated with indicated concentrations of ISO-1 for 24 hours. **(E)** Quantitative densitometric analysis of protein bands first normalized to GAPDH and then expressed as fold change relative to untreated controls. Graphs are expressed as the mean ± SD of each group from 3 independent experiments. *p < 0.05, and **p < 0.01 when compared with untreated controls.

The NF-κB and TNF-α signaling pathways are crucial elements in inflammation and tumorigenesis, as well as cancer cell proliferation and metastasis^28–31^. Furthermore, elevated TNF-α production by many cancer cells will positively feedback in an autocrine fashion to further augment the activation of the NF-κB signaling pathway to promote cancer cell proliferation, migration and invasion^29^. As MIF has been shown to activate NF-κB signaling and induce TNF-α production^32–35^, we thus examined the effects of ISO-1 on these two factors. To this end, we examined the mRNA and protein expression of MIF, NF-κB p65 and TNF-α using real-time PCR and immunoblotting respectively. As shown in Fig. 5A–C, when compared to untreated controls, ISO-1 treated cells exhibited significant and dose-dependent downregulation in the mRNA expression of MIF, NF-κB p65 and TNF-α. The change in MIF mRNA expression observed was consistent with the role of ISO-1 as an inhibitor of MIF expression. In line with reduced mRNA expression, the protein levels of NF-κB p65, pNF-κB p65, MIF and TNF-α were also similarly dose-dependently reduced in response to ISO-1 treatment (Fig. 5D,E). Phosphorylation of NF-κB p65 is necessary for the activation of NF-κB signaling and NF-κB-dependent transcriptional activities. Here we further showed that the phosphorylation of NF-κB p65 was attenuated when in the presence of ISO-1 suggesting the impairment of NF-κB signaling activation. Quantitative densitometric analyses confirmed the reduction in protein expression of TNF-α, pNF-κB p65, and NF-κB p65 (Fig. 5E). Together, data from our biochemical assays suggests that inhibition of MIF by ISO-1 impairs NF-κB signaling activation, and downregulates TNF-α gene expression and protein production in PANC-1 cells.

### ISO-1 suppressed tumour growth in PANC-1 xenograft mice model

**Figure 6:**
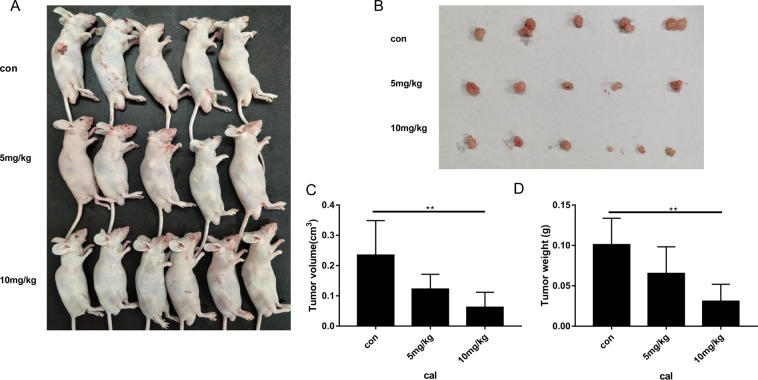
ISO-1-suppressed PANC-1 cell-induced tumor growth in xenograft mice model. (**A)** Tumour status *in vivo* in each group and **(B)** tumour tissues removed. **(C)** Tumour volume (cm^3^) and **(D)** tumour weight (g) were measured. Graph presented as mean ± SD; *p < 0.05 when compared to untreated controls.

With promising *in vitro* results, we next sought to investigate the potential therapeutic benefits of ISO-1 treatment on PANC-1 tumour growth in nude mice. Xenograft models were established by the subcutaneous injection of PANC-1 cells into the right axilla of nude mice. Tumours were allowed to proliferate and grow for two weeks after which mice were treated with intraperitoneal injections of low (5 mg/kg) or high (10 mg/kg) dose of ISO-1 for another 2 weeks (Fig. 6A). At the end of the experimental period, tumour tissues were excised (Fig. 6B) and tumour volume and weight were evaluated (Fig. 6C,D). As shown in Fig. 6A,B, the tumours from ISO-treated mice were significantly smaller than those from untreated controls. Quantitative measurement confirmed a dose-dependent reduction in tumour volume and weight when compared with untreated controls (Fig. 6C,D).

**Figure 7:**
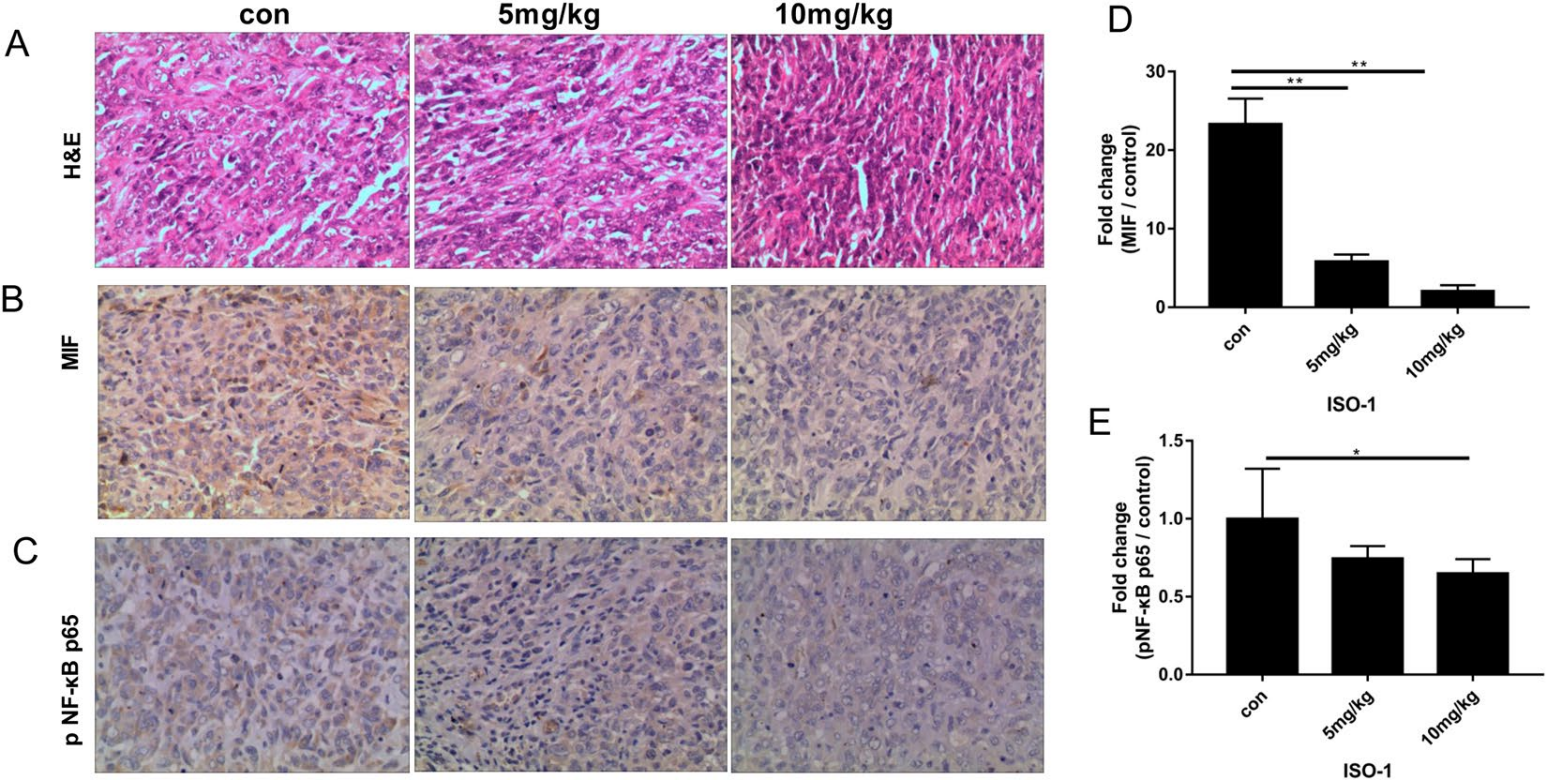
Expression of MIF and pNF-κB p65 were reduced in ISO-1 treated tumor tissues. Sectioned tissues were processed for **(A)** hematoxylin and eosin staining (H&E, magnification 200×), **(B)** MIF IHC (magnification 200×), **(C)** pNF-κB p65 IHC (magnification 200×). **(D,E)** MIF and pNF-κB p65 expression in ISO-1 treated tumour tissues were quantified and expressed as fold change relative to untreated controls; *p < 0.05 when compared to untreated controls.

Histological and immunohistochemical examination of tumour tissues further demonstrated that ISO-1 treatment significantly altered the cellular morphology of tumour tissue. Compared with the typical tumour morphology of high nucleus to cytoplasm ratio and numerous active mitotic cells seen in untreated samples, ISO-1-treated tumour tissues exhibited a less dense cell arrangement with cells appearing more flattened in morphology (Fig. 7A). Expression of MIF and pNF-κB p65 were also found to be markedly reduced in ISO-1-treated tumour tissues (Fig. 7B–E). These results were consistent with our *in vitro* cellular and biochemical analyses providing further evidence that ISO-1 can offer therapeutic benefits against the growth and progression of pancreatic cancer.

## Discussion

Macrophage migration inhibitory factor (MIF) a member of the tautomerase family of cytokines is highly expressed in a number of cancers including squamous carcinoma, glioblastoma, cervical adenocarcinoma, malignant melanoma, nasopharyngeal cancer, colon cancer, lung cancer, breast cancer, and prostate cancer^20,36–40^. Importantly, the expression of MIF is directly and positively correlated with the aggressiveness of the cancer phenotype. Our finding that MIF expression is substantially elevated in human para-carcinoma and pancreatic cancer tissues provides further evidence for the importance of MIF in pancreatic tumorigenesis. Thus, mounting evidence for the involvement of MIF in tumourigenesis makes MIF an attractive target for the development of therapeutic inhibitors. ISO-1 is a member of the isoxazoline class of compounds and considered the ‘gold standard’ archetypal competitive MIF inhibitor^15,24^. In this study, we demonstrated using *in vitro* cellular based assays that ISO-1 treatment inhibited PANC-1 human pancreatic cancer cell proliferation, migration and invasion. By real time PCR and western blot analyses, we further showed the downregulation of MIF, TNF-α and NF-κB p65 mRNA expression with concomitant reduction in their protein expression. Finally, we extended our *in vitro* work to an *in vivo* PANC-1 xenograft tumour growth model using BALB/c nude mice. Intraperitoneal treatment with ISO-1 markedly attenuated tumour growth, with significant reduction in tumour volume and weight, and downregulation of MIF expression in ISO-1 treated tumour tissues.

ISO-1 inhibits MIF’s tautomerase activity and also shown to prevent binding of MIF to its surface receptor thereby blocking MIF-induced signaling cascades^39^. In cancer, MIF predominantly signals through binding with the CD74 receptor, however, binding through the chemokine receptors CXCR2, CXCR4, and CD44 have also been shown^36,40^. Binding of MIF to CD74 activates several key signaling pathways including MAPK, PI3K/Akt and NF-κB, leading to cell proliferation and survival^41–43^. Interestingly, a recent study by Zheng *et al*. found that intracellular MIF itself can form a complex with the NF-κB p65 subunit and is necessary for the phosphorylation and nuclear translocation of NF-κB p65 in bone resorbing osteoclasts. Importantly, treatment with 4-IPP a suicide substrate/irreversible inhibitor of MIF attenuated NF-κB p65 phosphorylation^44^. Our results which showed downregulation of MIF, NF-κB p65 and pNF-κB p65 protein in PANC-1 cells is consistent with the work by Zheng and colleagues as well as other studies using ISO-1 in other cellular systems^45,46^.

As a pro-inflammatory mediator, MIF is capable of triggering via paracrine and autocrine loops, significant induction of pro-inflammatory cytokines such as IL-1β, IL-6, IFN-γ, and TNF-α^47,48^. In macrophages and RAW cells, ISO-1 was found to abrogate LPS-induced TNF-α production and secretion, and the genetic deletion of MIF in mice attenuates TNF-α production and protects against septic shock^49,50^. Our own biochemical analysis demonstrated that TNF-α gene and protein expression in PANC-1 cells were markedly reduced following ISO-1 treatment. In cancer cells, elevated production and secretion of TNF-α acts in a paracrine and autocrine manner to promote tumor proliferation, progression and ultimately metastasis^51–53^. In pancreatic cancer patients, serum levels of TNF-α have been shown to be substantially elevated^54,55^ providing further evidence for the intimate link between chronic inflammation and cancer development, with MIF being a likely candidate playing the role of the “middle man” bridging and intimately linking the two processes.

## Conclusion

A literature search identified three other studies examining the involvement of MIF in pancreatic cancer with two utilizing hamster MIF and cells as investigative models^21,22^, This study to our knowledge is the first study to investigate the effects of ISO-1 on human pancreatic cancer cells *in vitro* and pancreatic cancer cell-induced tumour growth *in vivo*. Our data have provided evidence that ISO-1 offers potential anti-cancer effects and further studies should be carried out to investigate the therapeutic application of ISO-1 or its derivatives for treatment of pancreatic cancer.

## Materials and Methods

### Human pancreatic cancer tumour samples

Tissue samples from patients with pancreatic cancer (n = 6) was obtained from the Department of Pathology of the First Affiliated Hospital of Zhengzhou University (Zhengzhou, China). The experiment has been approved by the Ethics Committee of Scientific Research and Clinical Experiment of the First Affiliated Hospital of Zhengzhou University (Ethics review number: 2019-ky-426) and was conducted in accordance with the relevant guidelines and regulations. The study is partly guided by the principles of the *Declaration of Helsinki 2018* (World Medical Association). Informed consent was obtained from all subjects. Samples were processed for hematoxylin and eosin (H&E) and immunohistochemical (IHC) staining as described below. The clinicopathological features are described in Table 1. All patients underwent surgery to remove cancer tissues including surrounding normal pancreatic tissue, tissue adjacent to carcinoma (para-carcinoma) and carcinoma tissue.

**Table 1:** Clinical characteristics of the pancreatic cancer patients.

Variable	characteristics
n	6
Gender (male/female)	4/2
Age (yrs)	(43~67)
Histologic diagnosis	Pancreatic Ductal Adenocarcinoma
TNM staging(n)	I (2) II (1) III (1) IV (2)

### Cell culture and ISO-1 treatment

The PANC-1 human pancreatic cell line was procured from FuHeng Biology (Shanghai, China) and cultured in RPMI 1640 medium (Gibco, Thermo Fisher Scientific, Waltham, MA, USA) supplemented with 10% fetal bovine serum (FBS; HyClone, GE Life Sciences, Chicago, IL, USA) and 100 units/ml penicillin and 100 μg/ml streptomycin. Cells were maintained in a 37 °C incubator with humidified atmosphere of 5% CO_2_. The MIF inhibitor, ISO-1, was purchased from Chengdu Purifa Technology Development Co. Ltd. (Chengdu, China) and dissolved in dimethylsulfoxide (DMSO; Sigma Aldrich, St Louis, MO, USA) to stock concentration of 100 mM and diluted further in media before use in downstream experimental applications.

### CCK-8 cell proliferation/cytotoxicity assay

We examined the effects of ISO-1 on PANC-1 cell proliferation using the Cell Counting Kit-8 (CCK-8) assay in accordance with manufacturer’s protocol (Beyotime Biotechnology, Jiangsu, China). PANC-1 cells were seeded into 96-well plate at density of 5 × 10^3^ cells/well in triplicates per treatment condition and cultured overnight. Cells were then treated with various concentrations (200, 400, 800, 1600 and 3200 μM) of ISO-1 for 48 and 96 hours. Cells treated with equal dilution of DMSO in media was used as untreated mock controls. In the last 4 hours of treatment, cells were incubated with 10 μl of CCK-8 reagent after which the absorbance at wavelength of 450 nm was measured on a microplate spectrophotometer reader (Varioskan LUX; Thermo Fisher Scientific). Cell viability was calculated as follows: [Abs_450_ of ISO-1 treatment group – Abs_450_ of blank]/[Abs_450_ of untreated controls − Abs_450_ of blank] × 100%. Blanks were empty wells containing media and CCK-8 reagent only.

### Cell-wound closure migration assay

The cell-wound closure migration assay was employed to examine the ability of PANC-1 cells to collectively migrate and recolonize the wound area following ISO-1 treatment. To this end, PANC-1 cells seeded in 24-well plates (density of 4 × 10^4^ cells/well) were cultured to 100% confluence after which a linear scratch wound was drawn down the center of the cell monolayer using the tip of a sterile pipette tip. Cellular debris and floating cells were removed, and fresh media supplemented with 2.5% FBS without or with ISO-1 (100 or 200 μM) were added to respective wells. Phase contrast images were taken immediately after the creation of the scratch wound and designated time point 0. Further images were captured at 24 and 48 hour time points. The width of the scratch wounds was quantified for each treatment condition at each time point using ImageJ software (NIH; Bethesda, MD, USA). The migration distance was calculated as follows: inhibitory distance (μm) = [wound width a time point 0 − wound width after ISO-1 treatment (at 24 or 48 hours)]/2. The change in migratory distance was then expressed as fold change relative to untreated controls; migration inhibitory fold = (average migration distance in the control group − average migration distance in the treatment group)/average migration distance in the control group.

### Transwell migration and invasion assay

The migration and invasion of PANC-1 cells in a 3-dimensional environment following treatment with ISO-1 were assessed using the Transwell Permeable Support Filter Chambers (8 μm pore size; Corning Inc, Corning, NY, USA) and BioCoat Matrigel Invasion Chambers (8 μm pore size; Corning Inc) respectively. PANC-1 cells were serum-starved for 24 hours prior to seeding (density of 3 × 10^5^ cells/well) into the upper inserts of the chambers in low serum (2.5% FBS) RPMI media. The bottom chamber wells were filled with high serum (15% FBS) RPMI media. Cells in the upper chambers were then treated without or with 400, 800, or 1600 μM ISO-1 for 24 hours. After the treatment period, the non-migrated/invaded cells remaining on the surface of the membrane in the upper chamber inserts were gently removed using a cotton-tipped swab, and migrated/invaded cells on the other side of the membrane or bottom surface of the insert were fixed in 4% paraformaldehyde (PFA). Fixed cells were then stained with 0.2% crystal violet and phase contrast images were captured under a light microscope. The number of migrated/invaded cells in 5 randomly-selected fields from each treatment condition were quantified using ImageJ and expressed as fold change relative to untreated controls.

### RNA extraction and quantitative real-time PCR (qPCR) analysis

Total RNA was extracted from PANC-1 cells treated without or with 200 or 400 μM ISO-1 for 24 hours using AxyPrep Total RNA Extraction Kit (Axygen Inc, Corning Inc) in accordance with manufacturer’s instructions. Complementary DNA (cDNA) was then synthesized using 2 μg of purified RNA using the BeyoRT II First Strand cDNA Synthesis Kit (Beyotime Biotechnology) and subsequently used as template for qPCR in reaction mixture containing specific forward and reverse primers, and TB Green Premix Ex Taq (Takara Bio Inc., Shiga, Japan). qPCR was performed on a Applied Biosystems ^TM^ Quant Studio^TM^3&5 (Thermo Fisher Scientific) with the following cycling conditions: 95 °C for 30 secs; followed by 40 cycles of 95 °C for 5 secs, 60 °C for 30 secs. Specific primers against the following human genes were used: *MIF* (Sense: 5′-GGACAGGGTCTACATCAACTA-3′, and Anti-Sense: 5′-TCTTAGGCGAAG GTGGAG-3′); *TNF-α* (Sense: 5′-TTATTTATTTACAGATGAATG-3′, and Anti-Sense: 5′-TTAGACAACTTAATCAGA-3′); *NF-κB p65* (Sense: 5′-CCTTATCAAGTGTCTTCCATCA-3′, and Anti-Sense: 5′-AATGCCAGTGCCATACAG-3′); and *GAPDH* (Sense: 5′-CTCTGGTA AAGTGATATTGT-3′, and Anti-Sense: 5′-GGTGGAATCATATTGGAACA-3′). The expression of target genes was normalized to internal housekeeping gene *GAPDH* using the 2^−ΔΔCT^ method.

### Protein extraction and immunoblotting

Total cellular proteins were extracted from PANC-1 cells treated without or with 200 or 400 μM ISO-1 for 24 hours using RIPA cell lysis buffer containing protease/phosphatase inhibitor cocktail. Protein lysates were cleared by centrifugation, concentration quantified, and then 20 μg of cleared proteins were resolved on 12% SDS-PAGE gel. Separated proteins were then transferred to nitrocellulose membranes (MilliporeSigma, Burlington, MA, USA) overnight at 4 °C. After blocking with 5% non-fat dry milk in Tris-buffered saline containing 0.1% Tween 20 (TBST) for 1 hour at room temperature, the membranes were incubated with primary antibodies against NF-κB p65, phospho-NF-κB p65 (pNF-κB p65) and GAPDH (1:1000 dilution in 1% non-fat dry milk in TBST; Abcam, Cambridge, UK) overnight at 4 °C. Membranes were extensively washed and then incubated with corresponding HRP-conjugated secondary antibodies (1:5000 dilution; Beyotime Biotechnology) for 1 hour at room temperature. HRP reactivity was detected by exposure to ECL Plus Chemiluminescent Detection System (Thermo Fisher Scientific) and imaged on a LI-COR Odyssey Imaging System (LI-COR, Lincoln, NE, USA). Densitometric quantitation was conducted for each protein band of interest using Studio Digits 3.1 software (LI-COR). The relative expression levels of target proteins were normalized to internal loading control GAPDH and expressed as fold change against untreated controls.

### PANC-1-induced tumour growth in nude mice xenograft model *in vivo*

Four-week-old male BALB/c nude mice were obtained from Beijing Vital River Laboratory Animal Technology Co. Ltd (Beijing, China). Animal experiments were approved by the Ethics Committee of Scientific Research and Clinical Experiment of the First Affiliated Hospital of Zhengzhou University (Ethics review number: 2019-ky-139; Zhengzhou, China). All experimental methods involving the use of animals were conducted in accordance with the animal experimental rules of the First Affiliated Hospital of Zhengzhou University. Mice received subcutaneous injections of 3 × 10^6^ PANC-1 cells in PBS (300 μl; 1 × 10^7^ cells/ml) into the right axilla of each mice. Two weeks after tumour cell injection, mice were randomly assigned to 3 groups with 6 mice per group. Control group was given intraperitoneal injections of normal saline, whereas treatment groups were given intraperitoneal injections ISO-1 at 5 mg/kg or 10 mg/kg, every day for 2 weeks. At the end of the 2 weeks treatment period, all mice were sacrificed and tumours were removed for measurement of volume (cm^3^) and weight (g). Formula for tumour volume was as follows: tumour volume (cm^3^) = [tumour length (cm) × tumour width (cm) × tumour height (cm)] × 0.52. The weight of the tumour tissues were measured using standard analytical laboratory balance. At the end of the experimental period, two mice exhibited tumour growth on their tail and hence removed from analysis.

### Histology and immunohistochemistry

Resected tumour tissues were fixed in 10% formaldehyde and then embedded in paraffin blocks for sequential sectioning into 4 μm thick sections. Tissue sections were processed for H&E staining as per our standard laboratory procedures. IHC staining was carried out using the High Efficiency Immunohistochemical Secondary Antibody Kit (Absin Bioscience Inc, Shanghai, China). Antibody against pNF-κB p65 was purchased from Cell Signaling Technology. The distribution and expression levels of MIF and pNF-κB p65 were statistically analyzed using ImageJ software, and the expression intensity was evaluated by the size of integrated optical density (IOD) value (IOD = average absorbance × area). The higher the IOD value, the higher the expression level of MIF and pNF-κB p65. Cells with higher expression of MIF and pNF-κB p65 was considered more mitotically active.

### Statistical analysis

Data presented in this study are expressed as mean ± standard deviation (SD) of at least 3 independent experiments unless otherwise stated. SPSS 17.0 software (IBM Corporation, Armonk, NY, USA) was used to determine statistical difference between groups using Student’s *t*-test or analysis of variance (ANOVA). Statistical difference was observed when calculated *p* was less than 0.05 or otherwise indicated.

## Supplementary information

Supplementary Information.
