# Supplementary material for: MIF inhibitor, ISO-1, attenuates human pancreatic cancer cell proliferation, migration and invasion in vitro, and suppresses xenograft tumour growth in vivo
Source: Sci Rep. 2020 Apr 21;10:6741. doi: 10.1038/s41598-020-63778-y (PMC7174354; doi:10.1038/s41598-020-63778-y)
Supplement: Supplementary file 1 — Supplementary Information. [file 41598_2020_63778_MOESM1_ESM.pdf]

MIF inhibitor, ISO-1, attenuates human pancreatic cancer cell proliferation, migration and invasion *in vitro*, and suppresses xenograft tumour growth *in vivo*

Bo Cheng [a,], Qiaofang Wang [a], Yaodong Song [a], Yanna Liu [a], Yanyan Liu [a], Shujun Yang [a], Dejian Li [a], Yan Zhang [a], Changju Zhu [a,*]

a Department of Emergency, the First Affiliated Hospital of Zhengzhou University, Henan 450052, China.

* Corresponding authors: E-mail: zhuchangju98@163.com.

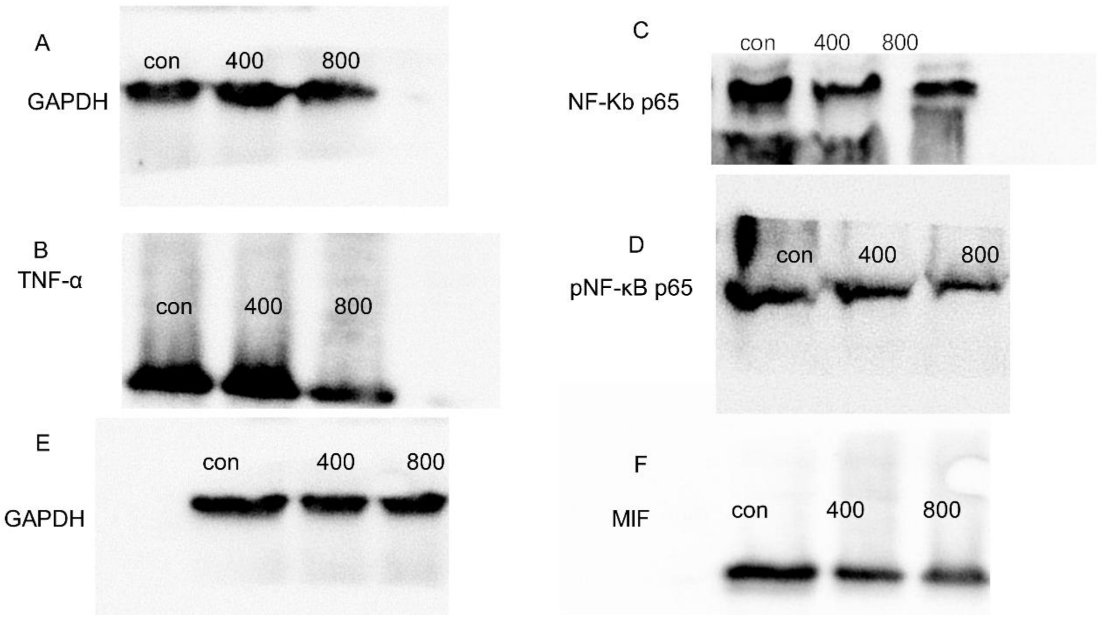

Supplementary Figure1. Full original western blot membranes for membrane strips presented in Figure 5 of the manuscript.
